# Cyclic Fatigue of Rotary Versus Reciprocating Endodontic Files: An In Vitro Study of Engine-Driven Endodontic Files

**DOI:** 10.3390/dj14040216

**Published:** 2026-04-08

**Authors:** Sverre Brun, Andrine Rebni Kristoffersen, Malene Nerbøberg Solsvik, Marit Øilo, Inge Fristad

**Affiliations:** 1 Department of Clinical Dentistry, Faculty of Medicine, University of Bergen, Årstadveien 19, 5009 Bergen, Norway; sverre.brun@student.uib.no (S.B.); marit.oilo@uib.no (M.Ø.); 2 Public Dental Health Service in Vestland, Årstadveien 21, 5009 Bergen, Norway; andrine.rebni.kristoffersen@vlfk.no; 3 Public Dental Health Service in Vestland, Gamle Dalavegen 15, 5600 Norheimsund, Norway; malene.nerboberg.solsvik@vlfk.no

**Keywords:** cyclic fatigue, reciprocation, rotation, endodontic instruments, NiTi

## Abstract

**Background/Objectives:** Instrument fracture remains a significant complication in endodontics. This study compared the resistance to cyclic fatigue failure between rotary and reciprocating nickel–titanium file systems, as well as differences related to file size and taper. **Methods:** Nineteen rotary and reciprocating file types (n = 10 per group) were evaluated in three independent test series, harmonized according to file size and system. Cyclic fatigue testing was conducted using a static model with a stainless-steel artificial canal, with an internal diameter of 0.9 mm, a 75° curvature angle, and a fixed radius for each series. Files were operated using preset programs on the X-Smart Plus, Rooter X3000, and Sendoline Endo torque-controlled motors. Time to fracture was recorded digitally, and the total number of full rotations to failure was calculated. The fractured fragments were examined with scanning electron microscopy and fractographic analysis. The data were analyzed using linear models in Stata version 19, with significance set at *p* ≤ 0.05. **Results:** Reciprocating file systems demonstrated greater time-to-fracture fatigue resistance than rotary systems. However, these differences were diminished or, in some cases, eliminated when normalized to the number of complete rotations. Fractographic analysis indicated that fractures predominantly resulted from tensile stress rather than shear forces. **Conclusions:** Reciprocating kinematics generally enhanced fatigue resistance compared with continuous rotation. The results suggest that fatigue resistance in machine-driven nickel–titanium instruments cannot be predicted by motion type or file design alone but reflects a complex interaction between alloy composition, heat treatment, and cross-sectional geometry.

## 1. Introduction

The development of root canal files has progressed substantially over recent decades, driven by innovations in both materials and instrument design. This evolution has highlighted the need for standardization of endodontic instruments [1,2]. Since 1985, technological advances, particularly in mechanical endodontics, have broadened the range of available instruments, with nickel–titanium (NiTi) systems becoming widely adopted due to their superior flexibility and strength compared with traditional stainless-steel alloys [3,4].

A central objective in root canal instrumentation is to enhance precision, efficiency, and safety. The introduction of NiTi instruments in the late 20th century marked a shift toward more standardized mechanical preparation techniques [3]. However, early generations of rotary NiTi systems exhibited high fracture rates, and multiple studies in the early 2000s documented frequent instances of instrument separation during clinical use [5,6].

In 2008, Yared introduced reciprocating kinematics specifically adapted for NiTi instruments, representing a significant conceptual and practical shift in canal preparation [7]. An important historical precursor to the modern reciprocating technique was the Giromatic system (Micro Mega, Besançon, France), developed in 1964 [8,9]. These reciprocating movements also simulate the motions used in the balanced force technique, later developed by Dr. Roane in 1985 [10]. Building on these foundations, Yared and colleagues refined reciprocating motion for NiTi instruments and helped establish its widespread clinical acceptance, leading to its growing dominance in contemporary endodontic practice.

Reciprocating motion alternates clockwise and counterclockwise rotations, reducing continuous loading on the file. By intermittently releasing stress, reciprocation may limit the risk of the instrument binding to canal walls, an issue more common in continuous cutting with purely rotary systems. This mechanism has been associated with increased resistance to both cyclic fatigue and torsional failure. In clinical settings, however, instrument separation is typically multifactorial, and debate persists regarding which failure mode is most common during canal shaping [11].

Cyclic fatigue occurs when an instrument undergoes repeated tension–compression cycles, particularly at the point of greatest curvature, eventually leading to material failure even under submaximal loads [12]. Torsional failure, in contrast, arises when the file tip or another segment becomes locked within the canal while the shank continues rotating, thus resulting in fracture once the applied torque surpasses the material’s torsional limit.

Contemporary innovations in endodontic instrument manufacturing, including heat treatment and surface modification, aim to reduce fracture risk and improve mechanical performance [13,14,15]. Many leading rotary and reciprocating systems now utilize controlled memory (CM) wire, a heat-treated NiTi alloy designed to increase flexibility and enhance resistance to cyclic fatigue. Both alloy composition and cross-sectional design are believed to significantly influence an instrument’s flexibility and fracture resistance [16,17,18,19]. Nevertheless, instrument separation remains a clinical concern, particularly in anatomically complex or curved canals.

To enable meaningful comparison between reciprocating and rotary kinematics, reciprocating motion can be normalized to full rotations. Under such normalization, the potential influence of taper design and instrument size on fatigue behavior becomes theoretically apparent. This relationship remains insufficiently explored in existing fatigue-testing protocols.

The aim of this study was, therefore, to compare the cyclic fatigue resistance of reciprocating and rotary NiTi file systems, measured both as time to fracture and as total number of rotations. Six reciprocating and five rotary size-matched systems were evaluated in a static model to assess differences in mechanical behavior. Additionally, the influence of instrument size and taper on fatigue resistance was examined by comparing three WaveOne Gold file sizes (Primary, Medium, and Large) and two Sendoline S3 instruments with different taper profiles. The null hypotheses were (1) no difference in cyclic fatigue resistance exists between rotary and reciprocating systems; (2) no difference exists among instruments of different sizes within the same system; and (3) no difference exists between instruments with different taper profiles.

## 2. Materials and Methods

### 2.1. Endodontic Files Included

A total of 190 files were included in this study. The sample was based on systems with available files in sizes #25 and #30. Each file system was recorded with its LOT number and consisted of a series of 10 instruments, commonly used in fatigue testing [12] (Table 1). Files from reciprocating (alternating to-and-fro motions) file systems (WaveOne Gold Primary, WaveOne Gold Medium, WaveOne Gold Large [Dentsply Sirona, Charlotte, NC, USA], Reciproc Blue R25 [VDW, Munich, Germany], R-Motion #25 and #30 [FKG, Crêt-du-Locle, Switzerland], One RECI #25 [Micro-Mega, Coltene, Besançon, France], Golden One [ORBIS, Shenzhen, China] and Sendoline S3 #2 and #3 [Directa AB, Upplands Väsby, Sweden]) were compared with rotary (continuous one-way rotation) file systems (Golden Taper #25 and #30 [ORBIS, Shenzhen, China], ProTaper Gold F2 [Dentsply Sirona, Charlotte, NC, USA], One Curve Mini #25 [Micro-Mega, Coltene, Besançon, France], HyFlex CM #25 and #30 [Coltene, Whaledent GmbH, Langenau, Germany], and Sendoline S3 #2 and #3 [Directa AB, Upplands Väsby, Sweden]). The overall sample and the individual series included instruments with distinct geometric designs (Table 1).

The files were operated using the manufacturer’s recommended handpiece and motor (X-Smart Plus, Dentsply Maillefer, Ballaigues, Switzerland; Rooter X3000, FKG, Crêt-du-Locle, Switzerland; or Sendoline Endo Motor, Directa AB, Sweden). Pre-set programs specific to each file system were used. File testing was performed in three independent series (Table 1).

Series 1 included five file brands, with each tested in sets of 10. This series comprised both rotary and reciprocating systems of the same size, as well as reciprocating files of different sizes.

Series 2 included seven #25 files, also tested in sets of 10, representing both rotary and reciprocating systems.

Series 3 included five #30 files tested in sets of 10, again including both rotary and reciprocating systems. Sendoline files were operated in both rotary and reciprocating modes according to the manufacturer’s recommendations.

### 2.2. Artificial Root Canal

**Figure 1:**
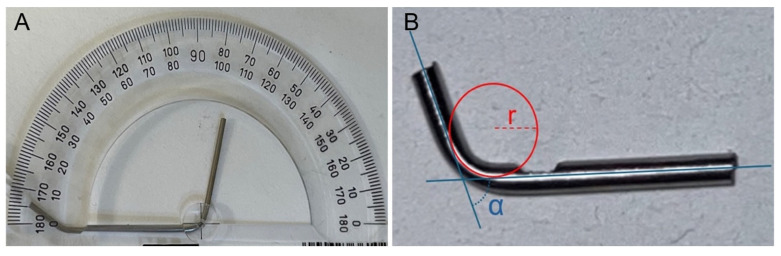
Manually fabricated simulated root canal (**A**), with defined angle (blue) and radius (red) (**B**).

Three artificial root canals with a curvature angle of 75° and an internal diameter of 0.9 mm were manually fabricated from a 17G medical stainless-steel syringe (Mediplast, Malmö, Sweden; LOT 191202 and 230903) (Figure 1). To maintain consistent testing conditions, one canal was assigned to each instrument series. The radius of curvature was measured individually for all canals. A 2 mm observation window was created in each canal to allow visualization of the apical 1 mm of the file tip during instrumentation. The distance from the point of maximum curvature to the file tip was approximately 5 mm. To reduce friction between the file and the metal canal, silicone spray (CRC Industries Europe, Zele, Belgium) was applied before each instrumentation.

### 2.3. Experimental Setup

**Figure 2:**
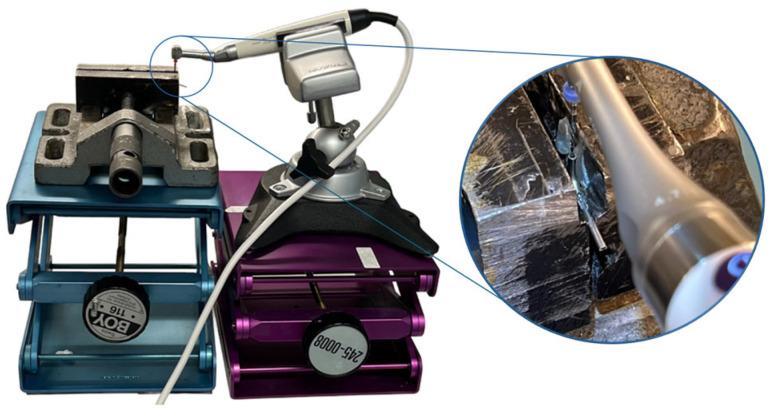
Setup for cyclic fatigue tests, with file in artificial canal (magnified to the left).

The experimental setup consisted of two bench vises, each mounted on an independent laboratory jack. One vise secured the artificial canal, while the other stabilized the handpiece containing the file (Figure 2). This configuration enabled controlled and reproducible vertical movement of the file into and out of the canal, while maintaining consistent alignment between all components throughout the experiments.

To minimize systematic bias from progressive wear of the testing tube, files were evaluated using a predefined symmetrical forward–reverse sequence. For each file system, two files were tested consecutively before proceeding to the next, and the sequence was repeated until all instruments had been assessed. The laboratory temperature was stable at 23 °C for the duration of this study.

### 2.4. Calculation of Full Rotations per Minute for Reciprocating Files

**Figure 3:**
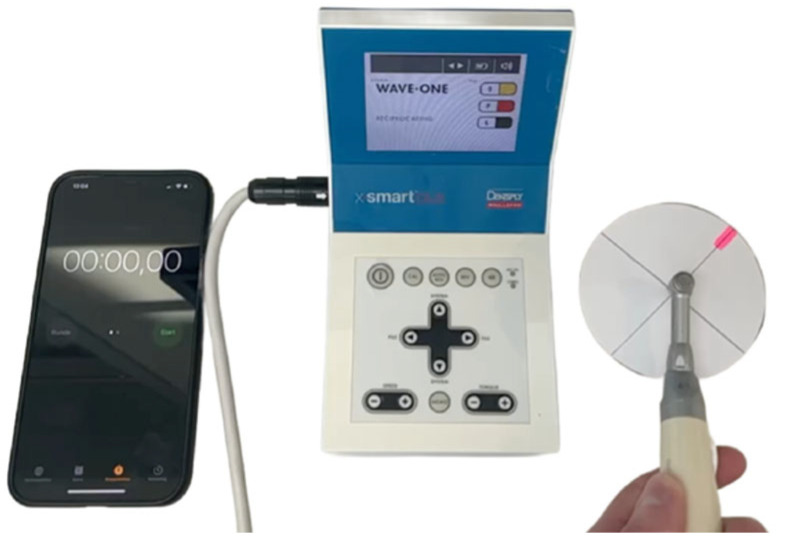
Setup for calculation of full rotations per minute for the reciprocating file systems.

Since most manufacturers of the reciprocating file systems do not specify the number of complete rotations per minute, the number of full rotations before fatigue fracture occurred was calculated for the different files. The same handpiece and corresponding preset for each file were used. A circular paper disc with a marked point was attached to a file and installed in the handpiece. The number of complete rotations per minute was determined by counting the rotations on a high-frame-rate video recording, which was analyzed by playback at reduced speed (Figure 3). Measurements were repeated twice by two independent observers.

### 2.5. Evaluation and Statistical Handling

The procedure was performed by the same operator within each series to minimize operator-related variability. A second operator recorded the time, which was stopped immediately upon file fracture. For each file, the time from initiation to fracture was measured and rounded to the nearest second.

The data (mean and standard deviation) were analyzed using linear models in Stata version 19 (StataCorp, College Station, TX, USA). Due to unequal variances (standard deviations) in the observations for the file types, robust variance estimates were applied. Post hoc analyses for pairwise comparisons were performed according to Scheffé. To check for the normality assumption, histograms and Shapiro–Wilk’s tests for normality were applied to the residuals for each model. The figures are presented with the standard error of the mean. The significance level was set at *p* ≤ 0.05.

### 2.6. Fractographic Analysis

One fractured file from each of the file systems was mounted in aluminum holders and subsequently analyzed using SEM (Phenom XL Desktop SEM) (Thermo Fisher Scientific, Waltham, MA, USA). The fractographic characteristics of the fracture surface and the outer surface near the fracture site were examined at different magnifications (×250, ×450, ×500 and ×1000).

Fractographic analysis was used to describe and compare the fractures that had occurred in the instruments.

## 3. Results

### 3.1. Artificial Canals

Although all three artificial root canals were constructed with the same curvature angle (75°), visual inspection and measurement revealed minor variations in their radii. The canal used for Series 1 had a radius of 5.5 mm, the canal for Series 2 had a radius of 5.8 mm, and the canal for Series 3 had a radius of 6.0 mm.

### 3.2. Full Rotations of Files per Minute

Two independent observers received identical results, with the number of full rotations per minute (the number of rotations, either in the clockwise or counterclockwise direction, depending on the system) for the different reciprocating file systems shown in Table 2. For comparison, the number of full rotations for the rotating instruments is included.

### 3.3. Cyclic Fatigue

#### 3.3.1. Series 1

- Fatigue fracture in seconds

**Figure 4:**
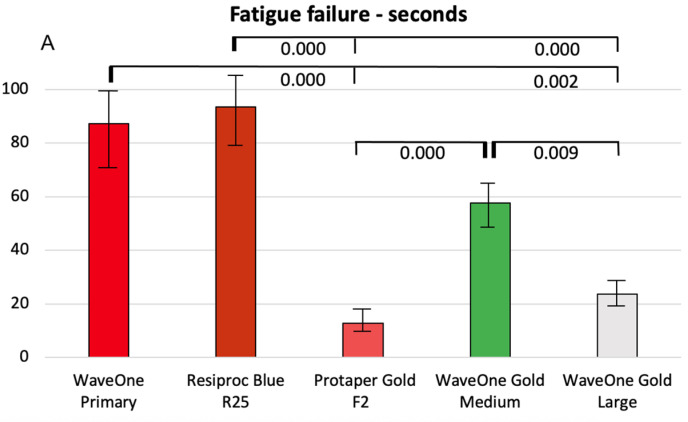

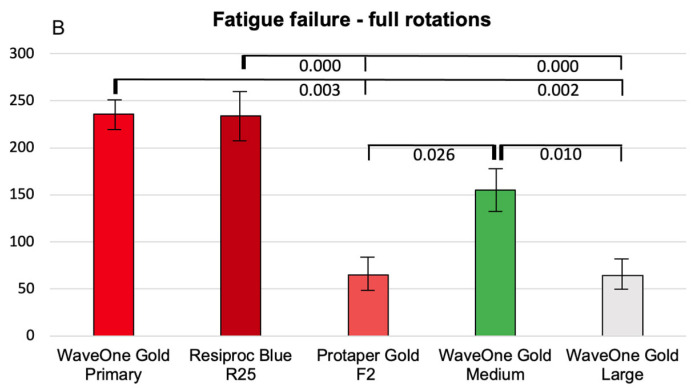
Cyclic fatigue in seconds (**A**) and full rotations (**B**), presented as the mean values with standard error of the means, along with significant differences between the groups. *p*-values are presented with 3 decimals.

Figure 4A presents the cyclic fatigue results, measured in seconds. No significant difference in time to fracture was observed between the two reciprocating systems, WaveOne Gold Primary and Reciproc Blue R25. In contrast, significant differences were found among the WaveOne Gold instruments of different sizes, with larger instruments exhibiting reduced resistance to cyclic fatigue. The rotary instrument ProTaper Gold also showed significantly lower resistance to cyclic fatigue compared with reciprocating instruments of the same size.

- Fatigue fracture in full rotations

Figure 4B presents the cyclic fatigue results expressed as the number of rotations. No significant difference was observed between the reciprocating files WaveOne Gold Primary and Reciproc Blue R25 in the number of cycles completed before fracture. However, significant differences were found among the WaveOne Gold files of different sizes, consistent with the results measured in seconds. The number of cycles to fracture decreased with increasing apical size. The rotary files also demonstrated significantly lower resistance to cyclic fatigue compared with reciprocating files of the same size.

#### 3.3.2. Series 2

- Fatigue fracture in seconds

**Figure 5:**
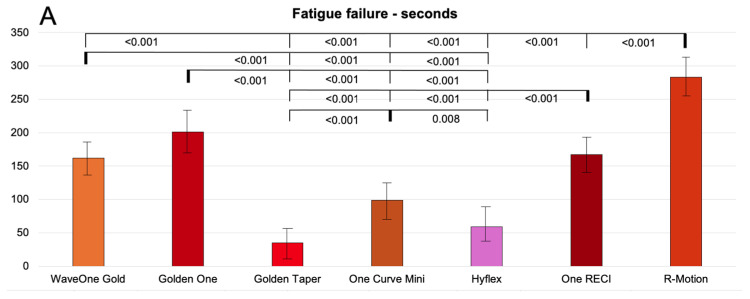

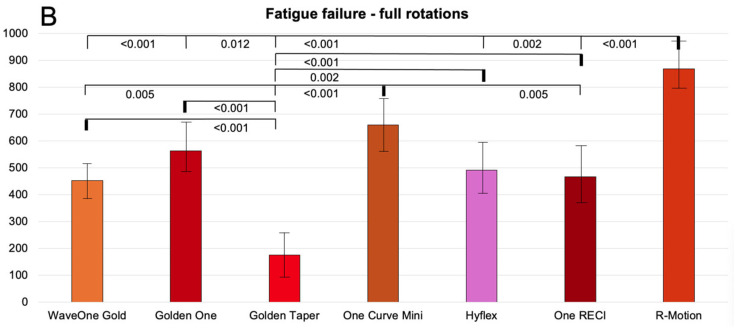
Cyclic fatigue of size 25 instruments in seconds (**A**) and full rotations (**B**), presented as the mean values with standard error of the means, along with significant differences between the groups. *p*-values are presented with 3 decimals.

Figure 5A presents the cyclic fatigue fracture results for size #25 instruments, measured in seconds. Overall, the reciprocating files demonstrated significantly greater resistance to cyclic fatigue than the rotary files. Among the rotary instruments, One Curve Mini showed the highest resistance, whereas R-Motion exhibited the greatest resistance among the reciprocating systems.

- Fatigue fracture in full rotations

Figure 5B presents the cyclic fatigue results expressed as the number of rotations for size #25 instruments. The findings were generally consistent with those obtained when fatigue was measured in seconds. However, for the rotary instruments One Curve Mini and HyFlex, the relative resistance increased when adjusted for the number of full rotations, as these systems operate at higher rotational speeds (400 and 500 rpm, respectively). When evaluated by the number of rotations, the rotating file One Curve Mini outperformed the reciprocating files WaveOne Gold and One RECI, the latter originating from the same manufacturer.

#### 3.3.3. Series 3

- Fatigue fracture in seconds

**Figure 6:**
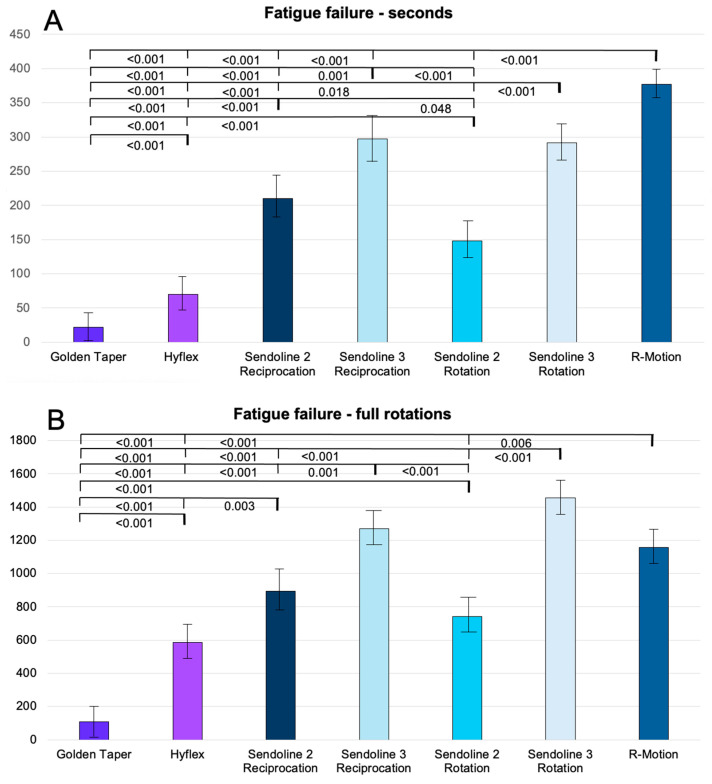
Cyclic fatigue of size 30 instruments in seconds (**A**) and full rotations (**B**), presented as the mean values with standard error of the means, along with significant differences between the groups. *p*-values are presented with 3 decimals.

Figure 6A presents the cyclic fatigue fracture results for size #30 instruments, measured in seconds. As in the previous series, the reciprocating files exhibited greater resistance to cyclic fatigue than rotary instruments, although some variation was observed among the different groups. Among the rotary systems, Sendoline demonstrated the highest resistance, whereas R-Motion showed the greatest resistance among the reciprocating files.

- Fatigue fracture in full rotations

Figure 6B presents the cyclic fatigue results expressed as the number of rotations for size #30 instruments. Compared with the results measured in seconds (Figure 6A), the influence of rotational speed becomes evident. When adjusted for full rotations, the rotary Sendoline S3 file demonstrated the highest relative resistance to cyclic fatigue. The HyFlex files also showed improved relative resistance when evaluated by the number of rotations rather than by time to fracture.

### 3.4. Fractographic Analysis

**Figure 7:**
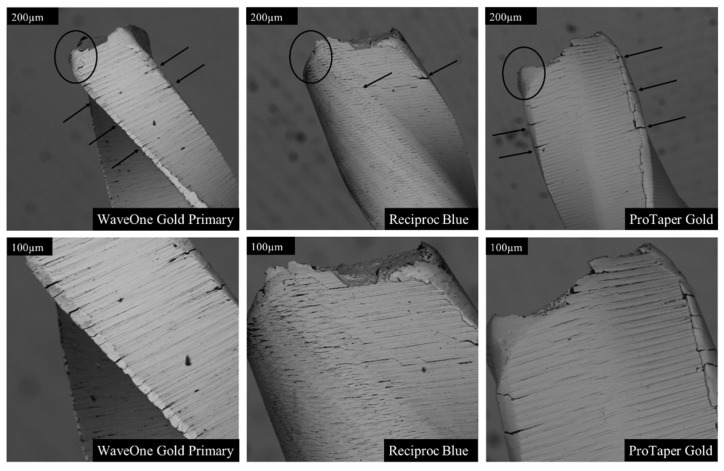
Lateral SEM views of three file systems tested in Series 1, shown at two magnifications. Notable differences were observed in the number and distribution of cracks along the cutting edges (black arrows), as well as in the distance between the final visible crack and the fracture plane. All instruments exhibited blunting of the cutting edges at the fracture surface (circled). Among the files, Reciproc Blue demonstrated the least wall deformation and the smallest degree of edge blunting.

**Figure 8:**
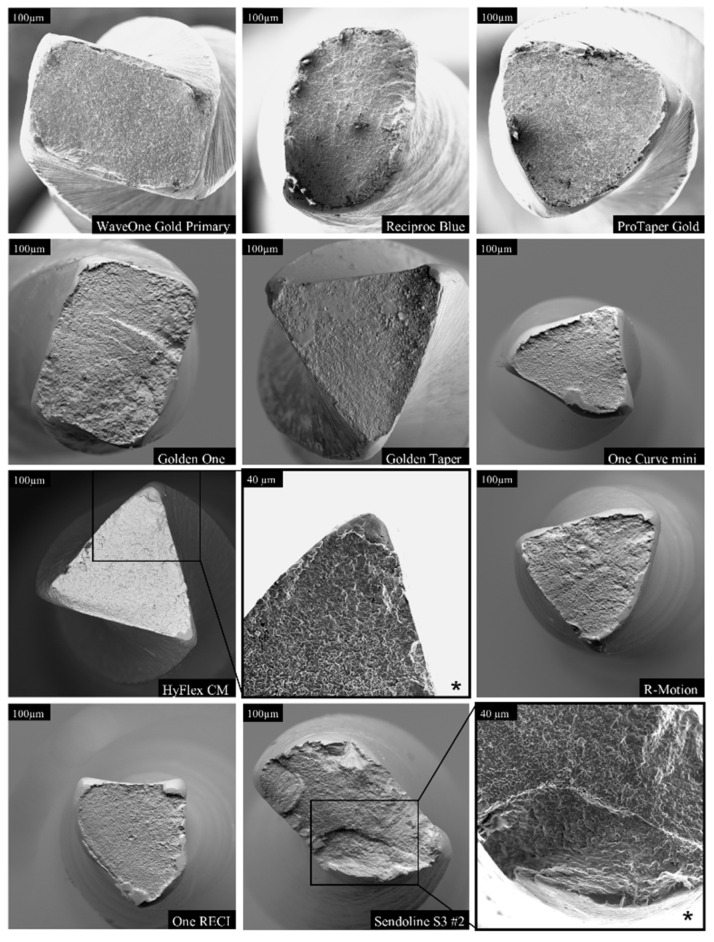
Comparison of fracture surfaces of all tested file systems at 450× magnification. The images illustrate clear differences in cross-sectional geometry and fracture surface morphology. Additional views at 1000× magnification (*) highlight the regions where material failure initiated. Cross-sectional area varied according to file size and the distance of the fracture plane from the instrument tip. Among the systems, the Sendoline files consistently exhibited a more granular and irregular fracture surface.

**Figure 9:**
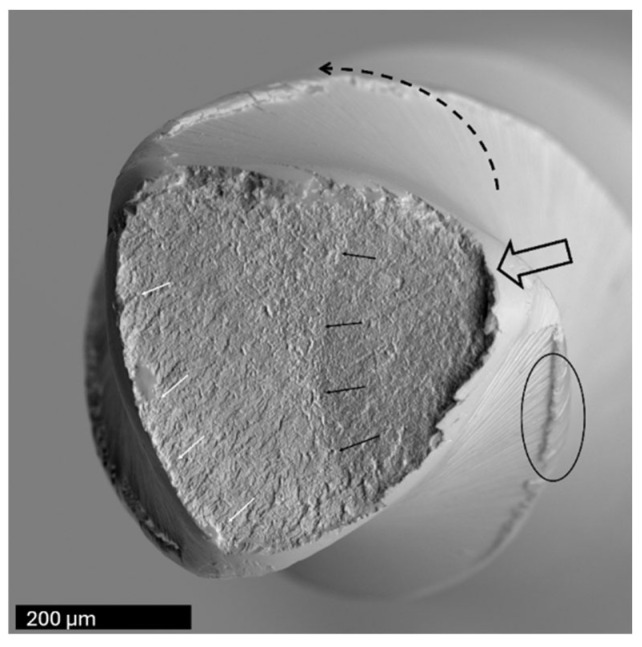
Scanning electron microscopy fractographic analysis of a ProTaper Gold instrument. The fracture originated at a corner of the triangular cross-section (open arrow) and progressed in a stepwise manner. A distinct arrest line is visible (small black arrows), marking temporary interruption of crack propagation. The final fracture advanced in the direction of instrument rotation (dashed arrow), as evidenced by river-mark patterns at the termination of the fracture surface (white arrows). Deformation of the cutting edge caused by rotational stress is also apparent (circled).

**Figure 10:**
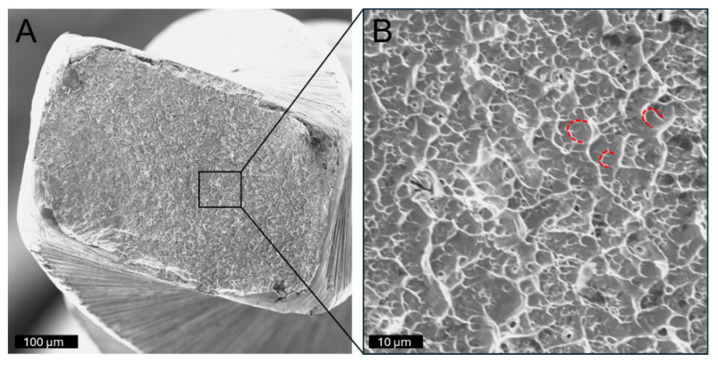
Fracture surface of a WaveOne Gold instrument showing characteristic features of ductile failure (**A**). Higher magnification of marked area shown in (**B**). Crescent-shaped depressions (dimples; dashed red lines) are evident across the fracture surface. These dimples exhibit a consistent opening direction but are not markedly elongated, indicating that tensile stress, rather than shear forces, was the primary driver of crack initiation and propagation.

The fractographic analysis revealed marked differences among the instruments in both fracture surface morphology and crack-propagation patterns (Figure 7, Figure 8, Figure 9 and Figure 10). Examination of the instrument surfaces showed distinct variation in structural features and wear, including differing degrees of crazing and chipping along the cutting edges (Figure 7). Micro-grooves oriented perpendicular to the long axis of the instruments were consistently observed. The degree and pattern of file twisting also differed between systems, both in geometry and in rotational density.

Fractographic characteristics further demonstrated variation in crystal structure across the tested instruments (Figure 8, Figure 9 and Figure 10). High-magnification analysis indicated that tensile stresses were the predominant factor contributing to fracture, as evidenced by classic tensile failure features on the fracture surfaces (Figure 8, Figure 9 and Figure 10).

## 4. Discussion

Endodontic treatment carries a risk of instrument fracture, which can compromise clinical outcomes [6,20]. File fracture mechanisms are primarily fatigue and torsional failure. This study evaluated cyclic fatigue fractures using a static, unrestricted rotation model to enable a controlled comparison of bending-related fatigue. The results showed considerable variation among instruments made from the same alloy but differing in size, geometry, and kinematics. NiTi alloys exhibit superior fatigue resistance compared with stainless steel, yet they show rapid crack propagation and minimal visible deformation before failure [5,21]. Their performance is influenced by stress- and temperature-dependent martensite–austenite transformations, and thermomechanical treatments that stabilize martensite improve flexibility and fatigue resistance [17]. Despite shared CM wire composition, SEM revealed clear differences in crystal morphology and fracture patterns, indicating that microstructure and processing play a greater role in fatigue resistance than nominal alloy composition alone [17]. Cross-sectional design also affects resistance, with triangular profiles outperforming rectangular ones in earlier studies [22].

The instruments tested represented rectangular, S-shaped, triangular/convex-triangular, and asymmetric designs. Fatigue resistance measured by time and rotations varied across series. Series 1 showed WaveOne Gold Primary and Reciproc Blue performing best; Series 2 showed R-Motion superior; and in Series 3, R-Motion performed best by time, while Sendoline S3 #3 produced the most rotations. S-shaped instruments performed best when included, whereas R-Motion performed best in their absence, indicating that design interacts with alloy treatment and kinematics to produce series-dependent outcomes.

Reciprocating files alternate between cutting and relief angles, whereas rotary systems use continuous rotation. These differences influence canal engagement, binding, and torsional loading [5,6]. The static stainless-steel canal eliminated dentin interaction and torque generation, meaning results reflect pure cyclic fatigue rather than combined fatigue–torsional failure [11,23].

When results were normalized to rotations, differences between reciprocating and rotary systems diminished or reversed. This demonstrates that rotational speed and choice of metric strongly influence system comparisons. Although reciprocating systems are commonly reported to have superior fatigue resistance, these comparisons are confounded by differences in canal geometry, curvature, temperature, and protocols [11]. Some rotary instruments (e.g., ProTaper F2) show improved fatigue resistance when used reciprocally [24]. In this study, reciprocating instruments generally performed best when evaluated by time, but these differences narrowed—and in Series 2 and 3 even reversed—when results were expressed as full rotations. For Sendoline S3, motion type did not significantly affect performance, suggesting that metallurgy and geometry may outweigh kinematics under static conditions [11,23].

Significant differences were observed within both the WaveOne Gold and the Sendoline S3 systems when comparing instruments of different dimensions. Instruments with larger cross-sections fractured earlier regardless of motion, reflecting greater stiffness and stress accumulation. Within Sendoline S3, instrument dimension—not motion—was the primary determinant of fatigue resistance. Larger instruments, therefore, do not improve cyclic fatigue resistance despite possible torsional advantages [25].

Differences in taper may influence fatigue resistance by altering mass at the curvature, although manufacturers specify taper only to 3 mm from the tip. Because D3 dimensions differed minimally, total mass at the bending point likely plays a more important role than nominal size.

SEM analysis showed fracture patterns consistent with ductile fatigue failure, including “dimples,” narrowing at the fracture site, and tensile-stress-associated cracks [16,26]. Reciproc Blue exhibited fewer surface defects than others in series 1, suggesting that smoother surfaces may delay crack initiation and enhance fatigue resistance.

### Methodological Considerations

Several methodological considerations must be acknowledged. Although natural teeth offer high clinical relevance, variations in anatomy, dentin hardness, and curvature limit standardization and comparability [27]. Because there is no universally accepted protocol for cyclic fatigue testing, inter-study comparison remains difficult [10,19]. Even in artificial canals with identical curvature, the effective bending radius depends partly on file size and stiffness, not only on canal shape [11].

Testing was performed using a static model without axial movement, which is known to underestimate fatigue resistance compared with dynamic models that distribute stress more evenly along the file [28,29]. Thus, the reported values should be interpreted as conservative estimates.

Temperature is another limitation. CM-wire instruments undergo temperature-dependent phase transformations [17]. In this context, the austenite start (As) and austenite finish (Af) temperatures are particularly relevant, as heating shifts the alloy toward a more austenite-containing phase [30,31]. At 23 °C, CM wire instruments may retain a martensitic character, with increased flexibility and greater resistance to cyclic fatigue. In contrast, at 35 °C, the same instruments may exhibit an altered phase balance, with increased austenitic content and reduced fatigue resistance [32]. Therefore, room-temperature testing may overestimate clinical performance. Transformation temperatures vary between brands, meaning comparative behavior may differ under real clinical conditions [33,34,35].

Three hand-shaped stainless-steel canals were used across the three series. Although each had a 75° curvature, small differences in radius and geometry limit direct comparison across series. This aligns with our results, showing a proportional increase in time to fracture, even with minor increases in canal radius between series.

Variations in file positioning within the test tubes may have influenced curvature engagement and the bending point experienced by the files. This variation may reflect differences in flexibility or file trajectory [36,37,38]. Thus, larger files showed lower fatigue resistance, suggesting increased fracture risk when the file is forced to follow the canal geometry. This interpretation may partly explain the significantly reduced fatigue resistance observed in larger files compared with smaller dimensions. Wear of the stainless-steel canals over time may have affected friction, although alternating the test sequence helped minimize this. Also, lubrication was provided using silicone spray rather than NaOCl or EDTA, and artificial metal or glass canals do not replicate dentin mechanics [11,25].

Despite these limitations, the model still supports valid internal comparisons. Small increases in canal radius produced proportional increases in time before fracture, confirming the sensitivity of cyclic fatigue to bending geometry. Within these constraints, instrument design, metallurgy, and dimensions appeared to influence fatigue resistance more than kinematics. Evaluating fatigue resistance in terms of completed rotations, rather than time to fracture, proved essential for comparing systems with differing rotational speeds.

## 5. Conclusions

Reciprocating systems generally demonstrated greater fatigue resistance than rotary systems when evaluated by time to fracture. However, differences decreased when results were normalized to rotations, showing that rotational speed strongly affects comparative outcomes. Fatigue resistance reflects an interplay of alloy treatment, heat processing, and cross-sectional geometry rather than motion type alone.

The hypothesis of no difference between file sizes was rejected, as larger instruments consistently exhibited reduced fatigue resistance. Across all series, S-shaped designs showed the highest resistance when present, while triangular designs performed best when they were absent. These findings highlight the dominant role of instrument dimensions, metallurgy, and cross-sectional geometry—over kinematics—in determining cyclic fatigue resistance.

## Figures and Tables

**Table 1:** Tested files with corresponding producer, movement, LOT numbers, taper, configuration, dimensions at D3 and series.

File	Producer	Movement	LOT-Number	Taper Apical 3 mm	Configuration 3 mm from Tip (D3)	Dimension D3	Series
WaveOne Gold Primary	Dentsply Sirona	Reciprocating	LOT 1764698	7%	Rectangular	0.46 mm	1
Reciproc Blue R25	VDW	Reciprocating	LOT 372277 (1–4), LOT 370203 (5–10)	8%	S-shaped	0.49 mm	1
ProTaper Gold F2	Dentsply Sirona	Rotation	LOT 1776409	8%	Triangular	0.49 mm	1
WaveOne Gold Medium	Dentsply Sirona	Reciprocating	LOT 1764698	6%	Rectangular	0.53 mm	1
WaveOne Gold Large	Dentsply Sirona	Reciprocating	LOT 1753289	5%	Rectangular	0.60 mm	1
WaveOne Gold Primary	Dentsply Sirona	Reciprocating	LOT 1753282	7%	Rectangular	0.46 mm	2
Golden One #25	ORBIS	Reciprocating	LOT 230508W02LN	7%	Rectangular	0.46 mm	2
Golden Taper #25	ORBIS	Rotation	LOT 23060107	8%	Triangular	0.49 mm	2
One Curve mini #25	Micro Mega	Rotation	LOT 714046	6%	Triangular	0.43 mm	2
HyFlex CM #25	Coltene	Rotation	LOT M78097	6%	Triangular	0.43 mm	2
One RECI #25	Micro Mega	Reciprocating	LOT 688328	6%	Asymmetric	0.43 mm	2
R-Motion #25	FKG	Reciprocating	LOT KP13, KG04	6%	Triangular	0.43 mm	2
Golden Taper #30	ORBIS	Rotation	LOT 23060107	9%	Triangular	0.57 mm	3
Hyflex CM #30	Coltene	Rotation	LOT M35592	4%	Triangular	0.42 mm	3
Sendoline S3 #2	Directa AB	Reciprocating	LOT 2310048	6%	S-shaped	0.48 mm	3
Sendoline S3 #3	Directa AB	Reciprocating	LOT 2310049	4%	S-shaped	0.42 mm	3
Sendoline S3 #2	Directa AB	Rotation	LOT 2310048	6%	S-shaped	0.48 mm	3
Sendoline S3 #3	Directa AB	Rotation	LOT 2310049	4%	S-shaped	0.42 mm	3
R-Motion #30	FKG	Reciprocating	IS17, JG19, JP21, HR02, KG04	4%	Triangular	0.42 mm	3

**Table 2:** Tested files with corresponding complete rotations per minute.

File	Complete Rotations Per Minute
**Reciprocating files**	
WaveOne Gold	162
Reciproc	150
R-Motion	187
Sendoline Reciprocating	256
One RECI	168
Golden One	168
**Rotating files**	
Golden Taper	300
Hyflex	500
ProTaper	300
Sendoline Rotary	300
One Curve Mini	400

## Data Availability

The raw data supporting the conclusions of this article will be made available by the authors upon request.

## Abbreviations

The following abbreviations were used in this manuscript:

EDTA	Ethylenediaminetetraacetic Acid
NiTi	Nickel–Titanium
SEM	Scanning Electron Microscopy
